# Bulk and Single-Cell Next-Generation Sequencing: Individualizing Treatment for Colorectal Cancer

**DOI:** 10.3390/cancers11111809

**Published:** 2019-11-18

**Authors:** Ioannis D. Kyrochristos, Demosthenes E. Ziogas, Anna Goussia, Georgios K. Glantzounis, Dimitrios H. Roukos

**Affiliations:** 1 Centre for Biosystems and Genome Network Medicine, Ioannina University, 45110 Ioannina, Greece; ikyrochristos@hotmail.com (I.D.K.); deziogas@hotmail.com (D.E.Z.); 2 Department of Surgery, Ioannina University Hospital, 45500 Ioannina, Greece; gglantzounis@gmail.com; 3 Department of Surgery, ‘G. Hatzikosta’ General Hospital, 45001 Ioannina, Greece; 4 Department of Pathology, Ioannina University Hospital, 45500 Ioannina, Greece; goussiaanna@gmail.com; 5 Department of Systems Biology, Biomedical Research Foundation of the Academy of Athens (BRFAA), 11527 Athens, Greece

**Keywords:** colorectal cancer, genomic and transcriptomic landscapes, intra-tumor heterogeneity, liquid biopsies, next-generation sequencing

## Abstract

The increasing incidence combined with constant rates of early diagnosis and mortality of colorectal cancer (CRC) over the past decade worldwide, as well as minor overall survival improvements in the industrialized world, suggest the need to shift from conventional research and clinical practice to the innovative development of screening, predictive and therapeutic tools. Explosive integration of next-generation sequencing (NGS) systems into basic, translational and, more recently, basket trials is transforming biomedical and cancer research, aiming for substantial clinical implementation as well. Shifting from inter-patient tumor variability to the precise characterization of intra-tumor genetic, genomic and transcriptional heterogeneity (ITH) via multi-regional bulk tissue NGS and emerging single-cell transcriptomics, coupled with NGS of circulating cell-free DNA (cfDNA), unravels novel strategies for therapeutic response prediction and drug development. Remarkably, underway and future genomic/transcriptomic studies and trials exploring spatiotemporal clonal evolution represent most rational expectations to discover novel prognostic, predictive and therapeutic tools. This review describes latest advancements and future perspectives of integrated sequencing systems for genome and transcriptome exploration to overcome unmet research and clinical challenges towards Precision Oncology.

## 1. Introduction

The validity of next-generation sequencing (NGS) at both bulk and single-cell levels in the identification of disease associated variants and tumor heterogeneity has transformed biomedical and cancer research [1,2,3,4,5,6]. The precise characterization of genetic heterogeneity with targeted NGS (tNGS) and whole-exome sequencing (WES), genomic variation with whole-genome sequencing (WGS) and transcriptional variability with RNA sequencing (RNAseq) and chromatin immunoprecipitation sequencing (ChIP-seq) has provided new knowledge on tumorigenesis, metastasis, drug response and relapse [7,8,9,10,11,12].

Although conventional research on the basis of tumor homogeneity and stability, as well as the linear single-gene transcription concept, has improved oncological outcomes for colorectal cancer (CRC) patients through the standardization of diagnosis, TNM staging and multimodal treatment, major clinical challenges remain unresolved [13,14]. Indeed, 50 years after the declaration of war on cancer [15] malignancy remains a principal cause of death in the industrialized world [16]. The valid identification of intra-tumor heterogeneity (ITH) at bulk and single-cell resolution, in conjunction with NGS of matched circulating cell-free DNA (cfDNA-NGS) are revolutionizing cancer science, creating new exciting opportunities to individualize therapy and substantially reduce oncological events [8,17,18,19]. Indeed, progress has been so rapid that these discoveries have already been translated into early-phase clinical trials [17,20]. Moreover, evidence on extensive inter- and intra-patient genetic, genomic and transcriptional heterogeneity [7,8,9] has underlined the need for combinatorial targeted therapy to improve the rates of disease-free, progression-free and overall survival [17,20,21,22]. This review discusses latest science advances, bottlenecks and future capabilities of genomic and transcriptomic landscape dissection in time and space towards precise prediction of drug response and tailored treatment for sporadic colorectal cancer.

## 2. Advances and Limitations in the Management of Colorectal Cancer

Colorectal cancer is currently the third most common cancer type, with over 1.8 million new diagnosed cancers, and the second leading cause of cancer-related mortality, accounting for over 881,000 deaths, in 2018 worldwide [16]. Considering the corresponding figures in 2012 (1.4 million cases and 693,900 deaths respectively), one can extract meaningful conclusions [23]. First, the substantial increase in incidence suggests lack of effective primary preventive strategy development, and second, despite a statistical survival improvement over time in the Western world [13], mortality/incidence ratio remains unchanged. These facts reflect the slow progress of conventional research and clinical practice regarding prevention and standardized multimodal treatment of CRC over the 50-year long war on cancer [15,24]. Nowadays, development of targeted therapies is still guided by the central dogma of molecular biology on linear transcription from single genes to mRNAs to proteins [24,25]. Moreover, all diagnostic approaches according to recent guidelines for treatment are based on pathohistological single-biopsy diagnosis, centered around the hypothesis of tumor homogeneity and stability [14].

Surgery aiming to complete tumor resection (R0) remains the fundamental principle of treatment with curative intent, even for patients with resectable liver and/or lung metastatic deposits, contrary to most other major cancer types [14,26]. Primary tumor localization in the colon or rectum is critical for therapeutic decision making. Adjuvant chemotherapy is indicated in most colon cancer patients [14]. By contrast, peri-operative treatment is suggested to most rectal cancer patients, including neo-adjuvant chemo-radiotherapy (NAC) followed by surgery and adjuvant chemotherapy (AC) [14]. However, an appropriate large-scale randomized controlled trial (RCT) has demonstrated no 10-year survival benefit from AC following NAC/surgery, particularly for patients with ypT0-2 disease (post-surgery staging after NAC) [27]. Thus, patients with ypT0-2 stage could potentially be spared the adverse effects associated with AC but further confirmation trials are required.

In contrast to modern non-targeted standard surgery, radiotherapy and chemotherapy, tumor-directed therapy with targeted drugs has recently surfaced as a rational hope to improve oncological outcomes of cancer patients [14]. However, the expectations of reducing relapse and cancer-related death rates through the addition of targeted agents to treatment regimens have not yet been met for resectable CRC. Indeed, high-quality evidence from RCTs suggests equal survival among resected stage II/III colon cancer patients treated with AC with or without targeted drugs [28,29], while for patients with resected CRC liver metastases progression-free survival was significantly shorter after the addition of cetuximab to standard AC [30].

Improvement of oncological outcomes via the standardization of multimodal treatment, including R0 surgery plus AC/NAC, has rather decelerated over the past decade [13,14,16,31], thus limiting expectations for further progress in the near future via conventional research-based clinical practice. An apparent innovative research strategy is primarily provided by latest scientific advances in genome and transcriptome exploration in time and space.

## 3. Next-Generation Sequencing: Progress from Static to Saptiotemporal Genomic and Transcriptomic Analyses

Next-generation sequencing (NGS) has brought a biotechnological revolution upon the study of genomic and transcriptomic variation, offering unprecedented accuracy, speed, and affordability at the same time. These advantages have led to the successful incorporation of NGS into the ENCODE [32], Mouse ENCODE [33] and modENCODE [34] projects exploring coding and non-coding genome functionality and gene expression regulation, as well as the exploitation of NGS systems within two very large-scale cancer consortia, namely The Cancer Genome Atlas (TCGA) [35] and the International Cancer Genome Consortium (ICGC) [36]. The applications of NGS span over several distinct sequencing systems. First, targeted NGS (tNGS) allows for rapid and accurate sequencing of a known-gene panel to detect specific cancer-related alterations at a very low cost, which has led to its widespread utilization in both laboratory and clinical settings. Second, whole-exome sequencing (WES) analyses the protein-coding region of genes accounting for 1.5% of the total genome, thus emphasizing on structural coding variants. Third, whole-genome sequencing (WGS) scans all coding and non-coding parts of the genome, enabling the identification of functional non-coding elements associated with disease. Fourth, RNA sequencing (RNAseq) or whole-transcriptome sequencing is used to characterize the structure and dynamics of the transcriptome and track gene expression with the capacity to discover novel molecular classifications [37,38]. Finally, chromatin immunoprecipitation sequencing (ChIP-Seq) enables genome-wide mapping of protein-DNA interactions by combining chromatin immunoprecipitation (ChIP) with NGS to identify the binding sites of transcription factors aiming to enhance our understanding of transcriptional regulation [39]. All methods have been extensively applied by numerous studies on both static and dynamic patient-derived samples to explore cancer evolution in time and space. Colorectal cancer represents a prime paradigm of explosive research progress, due to the high threat level it poses to public health on the one hand and the accessibility of both primary and metastatic tumor specimens on the other.

### 3.1. Single-Biopsy Genomics and Transcriptomics

Table 1 [7,40,41,42,43,44,45,46,47,48,49,50,51,52,53,54,55] summarizes most valid studies investigating genomic and transcriptomic colorectal cancer landscapes, as well as inter-patient heterogeneity, via the static analysis of single biopsies. Following the introduction of specific recommendations for large sample sizes to ensure the validity of genomic discovery of cancer genes and targetable mutations by Lawrence et al. in 2014 [7], several studies with significant samples sizes have emerged. Beyond confirmation of previous discoveries, numerous novel tumor-specific recurrently mutated and cancer driver genes were identified through whole-exome or genome analysis, both for sporadic as well as for familial CRC, including some that had not been implicated in any cancer type before, such as genes related to proliferation, apoptosis, genome stability, chromatin regulation, immune evasion, RNA processing and protein homeostasis. [7,42,43,51,52,55]. Apart from hypermutated tumors, comprehensive integrative analysis of CRC tumor/normal pairs revealed that cancers arising in the colon and rectum harbor highly similar genomic alterations, such as copy numbers, and most of the hypermutated tumors originate in the right colon [51]. Almost universal genomic events include activation of the WNT signaling pathway and inactivation of the TGF-β signaling pathway, leading to a subsequent increase of MYC activity, while genome changes often target the MAPK and PI3-K pathways but less frequently receptor tyrosine kinases [51]. Nevertheless, as not every mutation in driver genes is actually a driver itself, identifying cancer-drivers remains a key challenge, suggesting that, even though the expansion of the cancer driver gene corpus could be nearing the stages of completion, further systematic validation efforts are essential [52]. Additionally, high-volume research on rare variants associated with sporadic and familial CRC is still in its infancy, but promises to improve our understanding of the biological basis behind the disease and potentially inform future decision-making and drug development [42,43]. Quite notably, up to almost 75% of CRCs have been found to harbor potentially druggable single-nucleotide variants, indels or copy-number alterations [52], including targets within the WNT signaling, RTK/RAS and PI3K pathways [51] highlighting the utility and applicability of the basket design within patient-centric trials on targeted drug combinations.

Next-generation sequencing has also enabled the development of novel putatively clinically meaningful tumor molecular sub-classifications, particularly via gene expression analysis with RNAseq [41,46,50]. For instance, Guinney and colleagues reported a robust CRC classification into four distinct consensus molecular subtypes (CMS), based on findings from six independent classification systems, including RNAseq and other omics technologies [41]. The sub-classes and their distinguishing features include the microsatellite instability immune subtype (CMS1, 14%) which is hypermutated, microsatellite unstable with strong immune activation; the canonical subtype (CMS2, 37%), which is epithelial featuring marked WNT and MYC signaling activation; the metabolic subtype (CMS3, 13%), also epithelial with evident metabolic dysregulation; the mesenchymal subtype (CMS4, 23%), showing prominent transforming growth factor-β activation, stromal invasion and angiogenesis; and samples with miscellaneous features (13%), which possibly reflect transitioning states or ITH. Regarding distinct clinical characteristics CMS1 tumors were frequently in female patients, right-sided and of higher histopathological grade, CMS2 cancers were mostly in the left colon and CMS4 CRCs were diagnosed at more advanced stages. Prognosis-wise, the CMS4 subtype was an independent adverse prognostic factor for overall and relapse-free survival, the CMS2 subtype was a favorable prognostic marker and the CMS1 subtype correlated to very poor survival after relapse [41]. Moreover, a comparative analysis of molecular subtypes among gastrointestinal adenocarcinomas revealed at least a partial overlap with CMS classes, irrespective of primary cancer localization from esophagus to rectum [46]. Although a clinically optimal cancer sub-classification informing patient stratification for drug trials remains remote, the CMS molecular classification represents a comprehensive step-wise process and the most vigorous effort to date, with both prognostic and predictive relevance [41,50]. Beyond systematic CRC classification, other independent prognostic markers include several mutated genes [47,48] and primary tumor site of origin in the left or right colon associated with distinct genetic characteristics [44], while microsatellite instability has been confirmed as predictor of survival not only for CRC but across multiple other cancer types as well [40].

### 3.2. Bulk Inter- and Intra-Tumor Heterogeneity

Considering the simple acquisition of both primary and metastatic tumor specimens according to modern treatment guidelines, there have been several genomic studies investigating cancer heterogeneity both between and within tumors of the same individual, outlined in Table 2 [18,56,57,58,59,60,61,62,63,64,65,66,67,68,69]. Mutational concordance between matched primary and metastatic tumors was variable and dependable upon the sequencing method used, with exome and genome sequencing unveiling higher heterogeneity than tNGS [57,63]. For instance, computationally performed sub-clonality analysis has revealed that the number of sub-clones was highly consistent between primary tumor and matched metastasis [56]. Moreover, targeted sequencing of available gene panels has revealed high mutational consistency for key cancer-associated and driver genes, as for example KRAS, NRAS, BRAF, APC, PIK3CA and SMAD4, suggesting that driver events occur early in evolution and that a single biopsy of either tumor possibly accurately recapitulates the cancer mutational landscape for drivers of tumorigenesis [57,58,59]. However, data support a mutation type-specific model of heterogeneity. More specifically, single-nucleotide variants have been found as highly stable between tumors of a single patient, whereas copy number alterations featured high variance both between as well as within individual patients, following a spatial pattern [18,59]. Nevertheless, despite low overall discordance between matched tumors, incremental changes are observed probably due to co-evolution. This suggests that a static biopsy alone is likely sufficient in the chemotherapy-naïve patient, but additional dynamic biopsies of both primary tumor and metastases may be necessary to precisely tailor further treatment following drug resistance [60,61,62]. By contrast, a small WGS study supported a model of late dissemination, reporting that almost 40% of mutations are primary tumor- or metastasis-specific, identifying several metastasis-specific oncotargets as well, further supporting dynamic sampling over treatment to individualize and tailor therapy to account for tumor evolution [63].

In addition, distinct metastases have been found to harbor variant genomic architectures correlating to differential clinical outcomes, highlighting the potential to better optimize treatment via the molecular characterization of all patient tumors [69].

Intra-tumor heterogeneity was a universal finding, albeit at variable degrees. Hu et al., in a multi-regional WES study, revealed that both primary and metastatic tumors exhibited high levels of sub-clonality and ITH, indicating rapid temporal diversification [58]. In addition, the authors note that clonal evolution and selection is common at the early stages of tumorigenesis, contributing significantly to early dissemination and metastasis, and early identification of aggressive sub-clones could effectively guide more aggressive therapy [58]. Varying levels of intra-tumor heterogeneity of both primary tumors and metastases have also been demonstrated by several smaller studies integrating multi-regional sequencing [18,65,68], as well as via computational reconstruction of tumor phylogenetic trees [56]. Transcriptional ITH in space has also been identified, but validation will be required in larger studies to extract safe conclusions on its potential significance [66]. However, the evolutionary principles governing the life history of CRC leading to ITH remain unclear. Available data are highly controversial between linear [69], branching according to Darwin’s principles [61,67], and neutral [65] evolution, while some researchers propose a shift in the evolutionary history of CRC from Darwinian to neutral evolution during progression depending on tumor stage [58,64]. Taking into account the possibility of pre-existing minor aggressive sub-clones within the primary CRC [65] and the potential effect of the immune microenvironment on tumor evolution [69], further and more detailed exploration of cell-to-cell heterogeneity is required to delineate the complex mechanisms underlying tumorigenesis and metastasis

### 3.3. Liquid Biopsies: Early Diagnosis, Drug Response Prediction and Patient Monitoring

Lately, increasing interest has been concentrated on the evaluation of non-invasively acquired plasma samples, primarily aiming for biomarker identification and diagnosis through static NGS analysis cfDNA/ctDNA, as well as dissect dynamic cancer evolution and discover predictive markers via serial liquid biopsies over the course of therapy and during patient surveillance (Table 3) [19,22,70,71,72,73,74,75,76,77]. Apart from plasma cfDNA levels, which have traditionally been correlated to tumor burden, sequencing of cfDNA has been shown to accurately recapitulate the mutational landscape of the primary tumor [70]. More specifically, a very large-scale analysis on more than 20,000 oncologic patients with various late-stage cancers including CRC showed that tNGS of plasma cfDNA reliably detected tumor-derived alterations including major driver mutations, variants associated with drug resistance and clonal evolution in response to therapy as well as targetable mutations, detected in almost 20% of the total cohort [70]. Especially for colorectal cancer, Strickler et al. [19] reported that mutation frequencies identified by cfDNA-tNGS matched those of tumor sequencing studies and that tNGS liquid biopsies were capable of detecting alterations driving therapeutic resistance, potentially as a response to treatment. These results suggest the potential of large-scale tNGS-based liquid biopsies to discover novel diagnostic, prognostic and predictive biomarkers or validate findings from smaller studies [73]. Quite notably, the use of ctDNA mutational profiles as predictive biomarkers has already entered the early clinical trial setting, and early results have showed that patient stratification and treatment with drug combinations matched to the individual circulating variability is a promising strategy to achieve disease control, following confirmation [22]. Regarding early diagnosis, despite moderate results in stages I-II from early efforts such as the CancerSEEK test [72], highly encouraging results have only recently been reported as an initial preliminary analysis of the Circulating Cell-free Genome Atlas (CCGA) (NCT02889978), a very large-scale clinical trial aiming to enroll 15,000 participants evaluating the diagnostic utility of targeted, whole-genome and whole-genome bisulfite sequencing of cfDNA [71]. For 12 major causes of cancer-related mortality, CRC among them, sensitivity was 77% and 84% for stages II and III respectively, although sensitivity was only 34% for stage I tumors. Detection rates for CRC in specific were up to 74% for stages I-III combined. Thus, final results are eagerly anticipated to determine the true diagnostic power of the blood test in the screening setting [71].

Temporally collected serial liquid biopsies via cfDNA-NGS have been employed on the other hand in the search for a non-invasive tool enabling patient monitoring and potentially informing therapeutic decision-making. Earlier studies have already demonstrated the accuracy of cfDNA-NGS to identify tumor-specific mutations and correlated a reduction of cfDNA levels over therapy to clinical response and favorable outcomes [76]. Nevertheless, the exploration of spatiotemporal clonal evolution and the evaluation of emergent heterogeneity as a predictive biomarker via serial blood samples has only recently surfaced. For instance, Peeters and colleagues, in the ASPECCT study cohort of 261 patients with metastatic CRC, applied tNGS on cfDNA samples before and after panitumumab therapy to assess the predictive power of acquired RAS mutations [74]. Over anti-EGFR treatment approximately 20% of patients with wild-type KRAS at baseline featured emergent KRAS mutations as a response to therapy, often featuring multiple mutations in the same gene due to clonal evolution, highlighting a potential mechanism of acquired drug resistance. However, the authors underline that alternative drivers of resistance may exist outside the EGFR pathway, which were not captured by the limited 63-gene panel used in this study [74]. By contrast, a cfDNA-tNGS analysis conducted on a similar ASPECCT study subpopulation, despite noting the capacity of liquid biopsies to effectively monitor dynamic clonal cancer evolution, found that emergent RAS mutations lacked any association with patient outcomes and cannot be used as a predictive marker guiding decision-making on treatment, thus leaving room for controversy [75]. Furthermore, a smaller study demonstrated the capacity of serial liquid biopsies to track tumor dynamics and clonal responses to matched targeted therapy and predict time to disease progression, suggesting both prognostic and predictive utility [77]. Large-scale validation studies integrating much wider gene panels are hence required to establish the potential clinical applicability of serial NGS-based liquid biopsies for patient monitoring to readily predict therapeutic failure and disease relapse.

### 3.4. Spatiotemporal Intra-Patient Heterogeneity

On this basis, combined analysis of spatiotemporally collected matched tumor and plasma samples to identify intra-tumor and circulating heterogeneity respectively, which collectively comprise comprehensive intra-patient heterogeneity (Table 4) [17,20,78,79,80,81,82] currently represents the most promising strategy to explore tumor dynamics and identify markers predictive of drug resistance. One of the first efforts of comparative NGS analysis was performed by Siravegna et al. on 100 CRC patients [78]. Analysis of matched tumor and ctDNA during anti-EGFR targeted treatment uncovered mechanisms of secondary resistance to EGFR blockade and concluded that liquid biopsies could be a more robust alternative to tissue to track the genomic evolution of advanced CRC [78]. Concordant results have been reported for CRC under HER2 targeted therapy, namely trastuzumab and lapatinib. Most of the patients harbored drivers of resistance in plasma ctDNA and ctDNA-tNGS, such as mutations in ERBB2, RAS and PIK3CA, while liquid biopsies could detect therapeutic resistance with a sensitivity of over 85%. Moreover, comparisons between the mutational landscapes of distinct metastases from a single patient revealed distinct evolutionary mechanisms and drug sensitivity profiles, suggesting that, contrary to current practice, serial plasma samples as well as multiregional sampling of both primary and all matched metastatic tumors is required to optimize treatment according to the integrated intra-patient mutational landscape [79]. Intra- and inter-lesion heterogeneity of resistance mechanisms has also been demonstrated by several small-scale studies, highlighting the necessity of appropriately designed spatiotemporal genomic trials to systematically explore dynamic tumor evolution and identify robust predictive biomarkers [80,81,82].

As a result, the first early clinico-genomic trials are only just surfacing. The I-PREDICT study has already put the current paradigm of Precision Oncology trials to question, suggesting that individualized multi-drug targeting, as opposed to standard targeted monotherapies, sets the stage for future patient-centric trials [20]. Targeted NGS of both tumor and matched ctDNA samples successfully informed therapeutic decision-making in a large fraction of patients with various refractory cancers within a small cohort, improving disease control and survival rates and, in fact, in a pattern proportionate to the number of drugged alterations [20]. 

Moreover, Khan et al. [17] demonstrated the capacity of spatiotemporal multi-regional primary and progressive/metastatic tumor sampling, complemented by frequent serial cfDNA-NGS liquid biopsies during therapy, to predict time to therapeutic resistance and subsequent treatment failure, preceding clinical diagnosis, limiting however their primary scope on the RAS pathway [17]. Despite weaknesses, including the implementation of targeted NGS of a known gene panel, the focus on specific signaling pathways, the small sample sizes as well as the lack of a strict protocol, these studies represent early steps in the direction of spatiotemporal clinico-genomic studies exploring tumor evolution and potential implications of dynamic tumor heterogeneity for the clinic.

### 3.5. Translational Implications of Cell-by-Cell Cancer Variability

Multi-regional tumor profiling has indeed significantly advanced our understanding of intra-tumor heterogeneity and how it affects therapeutic response, cancer progression, metastasis and relapse. However, bulk genomic and transcriptomic profiling of a tumoral sample carries out only average measurements of cellular characteristics, thus masking critical aspects of ITH, such as rare resistant or aggressive cell subpopulations. Therefore, single-cell genomics and transcriptomics have surfaced as powerful approaches making possible the full and precise exploration of cellular properties at the level of individual cells [83]. Recent single-cell sequencing studies on CRC and their potential translational relevance are delineated in Table 5 [8,84,85,86,87,88]. Single-cell RNAseq has successfully improved upon existing CRC molecular classifications, via detecting distinct sub-clones within a single subtype previously identified through bulk tanscriptomics, with putative prognostic significance [85]. Additionally, single-cell multi-omics approaches have been utilized to trace epigenomic and transcriptomic dynamics of CRC, as well as potentially clinically relevant cell sub-clones associated with cancer progression and metastasis [84]. Most notably, an integrative single-cell analysis by Roerink and colleagues has revealed markedly differential responses to anti-cancer drugs owing to dynamic evolution, even between spatially and molecularly closely related cells within the same tumor [8]. These, along with further smaller studies [86,87,88], demonstrate the unprecedented power of integrated NGS systems to study the dynamics of the hallmarks of cancer and future research is eagerly anticipated to advance our understanding of tumor biology, as well as the origin and true phenotypic impact of cell-to-cell genomic, transcriptomic and epigenomic intra-tumoral variability.

### 3.6. Functional Non-Coding Mutations and Regulatory Network Exploration

It has been now well established that more than 90% of variants associated with common diseases, including cancer, lie within the non-coding areas of the genome [89]. However, the effects of non-coding sequence alterations on gene expression remain poorly understood [45]. Beyond genome-wide association studies [89], modern genome and transcriptome sequencing analyses have identified recurrent non-coding aberrations affecting regulatory circuitry in colorectal cancer, with putative prognostic significance [49,63]. Moreover, innovative research has identified regulatory networks comprised by several non-coding elements, mutations in which deregulate the expression of target genes irrespective of tumor type, some of which have not been identified as cancer drivers [45]. Therefore, future research is greatly motivated to better characterize the architecture of transcriptional networks and the regulation of gene expression and further elucidate on the role of non-coding functional mutations in cancer.

## 4. Future Perspectives

In the era of genome and transcriptome exploration in time and space towards Cancer Precision Medicine, NGS systems have a pivotal role in overcoming cancer challenges. Intra-tumor heterogeneity at both bulk and single-cell resolution has emerged as a prognostic and predictive biomarker, potentially informing individualized therapy. Moreover, simultaneous NGS of matched serial cfDNA/ctDNA could further increase the accuracy of drug response prediction.

Another significant NGS implication is the capacity to discover new druggable mutations [52,90], to expand the list of approved targeted drugs, addressing extensive genetic, genomic and transcriptional heterogeneity. On this basis, the selection of most effective drug combinations matched to the comprehensive intra-patient heterogeneity, combining intra-tumoral and circulating variability, could allow for optimal individualized therapy. However, these promising data call for validation before wide clinical implementation.

### 4.1. Emerging Clinical Trials in Precision Oncology

Early cancer diagnosis is the most crucial independent factor for improved overall survival, or even reaching cure. Regarding CRC in particular, survival after treatment for localized disease is approximately 90% [13]. Although colonoscopy remains the gold standard for early diagnosis of CRC, the available figures for cancer statistics for the United States suggest no increase in localized disease detection rates over the past decade [13,91]. Apparently, compliance for asymptomatic individuals over 50 years old remains quite low. Therefore, the discovery of a non-invasive test could dramatically increase early diagnosis. A rational research strategy has focused on the development of blood test screening tools. Next-generation sequencing of circulating cfDNA/ctDNA has already returned promising data, but the minute amounts of cfDNA associated with small primary tumors remains challenging to detect. Several studies have recently been published, but only two have provided exciting findings. First, the underway large-scale clinical trial “The Circulating Cell-free Genome Atlas” (NCT02889978) has reported encouraging early results on 1,422 patients with various cancer types, including CRC. Sensitivity for stage I-III CRC was 74%, but since most patients had stage II-IV cancer, the final results should be anticipated to assess the true efficacy of the test in early-stage, asymptomatic individuals [71]. And second, much more groundbreaking data on early non-invasive diagnosis have been reported by Cristiano et al. [29]. More specifically, sensitivity for stages I-II of several cancer types, including CRC, was 79% and the combination of targeted and whole-genome sequencing with machine learning achieved overall detection rates of over 90%, highlighting the potential for blood screening following validation clinical trials [29].

**Figure 1:**
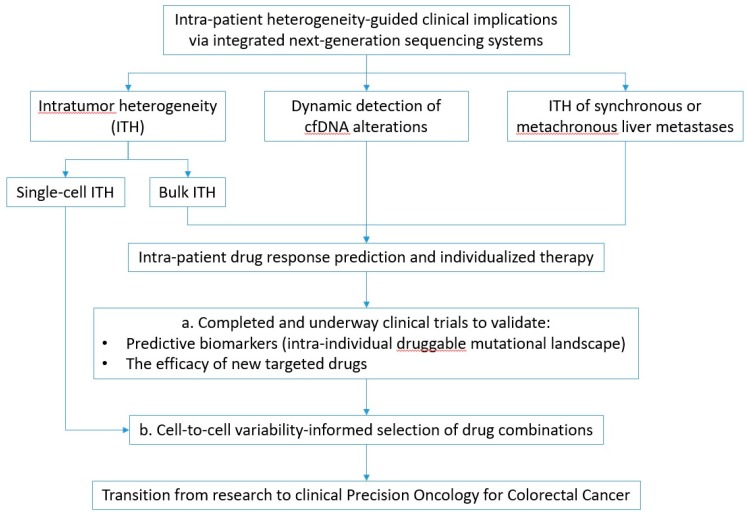
Putative clinical implications emerging from the breakthrough exploration of intra-patient intratumor and circulating heterogeneity. (a) Step-wise delineation of translational and clinical implications via genome and transcriptome sequencing. (b) Medium-term clinical expectations: Progress from genomic and transcriptomic studies to sequencing of bulk multi-regional primary and metastatic tumor tissue and matched serial cfDNA within appropriately designed clinical trials promises to realize the initial phase of Precision Oncology. Innovative future translational research: Emerging advances in single-cell exploration of genomic and transcriptional heterogeneity could enable the precise selection of drug combinations.

Rapid advances in NGS, particularly tNGS on a panel of known genes, have already led to the completion of early-phase trials [17,20,21,22], with more still underway [92]. However, despite promising published data on survival benefits via drug combinations within the basket trial design, further randomized controlled trials are required to draw definitive conclusions. Moreover, fundamental evidence on dynamic genomic clonal evolution [93] has created major clinical expectations for genomic trials of spatiotemporal design [94] unraveling the clinical utility of ITH and matched NGS of serial liquid biopsies comprising comprehensive dynamic intra-patient heterogeneity [95]. This sophisticated clinical trial framework could not only validate the predictive capacity of intra-tumor and matched circulating mutational landscape, but also support the evidence-based discovery of novel oncotargets and drugs [96]. In Figure 1 we propose a step-wise approach to validate prognostic and predictive biomarkers, as well as new biomarker-directed drugs.

### 4.2. Pharmacogenomic Predictions at the Single-Cell Level: A New Horizon for Cancer Precision Medicine

Tremendous progress from bulk multi-regional tissue NGS to genome and transcriptome sequencing of hundreds of thousands single cells has opened new horizons towards the realization of the long-term researchers’ dream to accurately predict response to multiple drug combinations, including agents targeting both cancer and stromal cells [8,97]. Considering the hundreds millions of malignant and non-malignant cells comprising a solid tumor, recent successful exploration of the transcriptional landscapes of two million individual cells applying machine learning algorithms [2] highlights the future feasibility of full cell-by-cell intra-tumor variability characterization integrating artificial intelligence, towards individualized therapeutic response prediction.

More recently, pioneering technological combinations of single-cell NGS, CRISPR-Cas and Hi-C is transforming biomedical research, raising high hopes for understanding linear and non-linear interactions controlling gene expression at single-cell resolution [98].

### 4.3. Transcriptional Networks and Pharmaceutical Controllability

Evidence from genome-wide association studies on most disease- and cancer-associated variants residing outside of protein-coding genes [89] and the ENCODE project on the functionality of most of the non-coding genome [32] guides molecular fundamental research towards understanding regulatory networks and gene expression control. Recent studies implementing breakthrough combinations of WGS to identify functional non-coding mutations, RNAseq, ChIP-seq, Hi-C, genome/transcriptome engineering and single-cell NGS have provided deeper insights into regulatory mechanisms governing the expression of interacting genes [98,99,100,101]. Despite this groundbreaking progress, further sophisticated research is needed to accurately characterize driver alterations and key regulators of transcriptional networks, towards the controllability of aberrant dynamic linear and non-linear circuitry with next-generation therapies [102,103,104].

## 5. Conclusions

The validity of NGS systems in the exploration of genome- and transcriptome-wide heterogeneity is transforming biomedical and cancer research and highlights the new era of patient-centric genomic trials. Evidence-based clinical treatment with drug combinations within tNGS-based basket trials has already provided promising results. Ongoing and future spatiotemporal genomic and transcriptomic trials evaluating and potentially validating bulk ITH and matched cfDNA variability could establish both the discovery of new targeted drugs and the development of predictive biomarkers for individualized drug sensitivity-directed therapy. With a more distant perspective, progress in cell-to-cell heterogeneity-guided drug selection could enable optimized multi-targeted therapy matched to the comprehensive intra-patient druggable mutational landscape.

## Figures and Tables

**Table 1:** Exploration of inter-patient genomic and transcriptional heterogeneity.

Patients/Samples	Technology	Findings and Potential Clinical Implications	Ref.
5930 (18 cancer types)	WES	MSI-positive tumors were found in 14/18 cancer types and MSI had prognostic significance	[40]
4151	RNAseq, Affymetrix and Agilent gene expression platforms	Four consensus molecular subtypes were identified potentially informing patient classification	[41]
1439	WGS	40 new independent association signals were discovered prompting further research for rare variants	[42]
1006 (familial)	WES	16% of familial CRCs had highly penetrant rare mutations including 3 novel candidate cancer driver genes (*POT1*, *POLE2*, *MRE11*)	[43]
999 (601 PTs, 533 MTs)	tNGS	Right- and left-sided CRCs harbored distinct oncogenic mutations, potentially explaining differences in survival	[44]
930 from 22 cancer types	WGS, RNAseq	A network of 193 non-coding loci was identified, affecting gene expression and warranting further research on functional mutation significance	[45]
921 (multiple GI cancer types)	WES	5 major GI adenocarcinoma subtypes were identified, with potential prognostic relevance	[46]
511 from QUASAR 2 trial	tNGS	*TP53*, *KRAS*, *BRAF* and *GNAS* mutations were independent adverse prognostic factors and total mutation burden correlated with favorable survival, while MSI was not associated with survival	[47]
468	tNGS (1,321 gene panel)	17 genes correlated to prognosis and absence of *APC* mutations was associated with worse prognosis	[48]
341	RNAseq	20 dysregulated lncRNAs were identified, potentially related to tumorigenesis and/or progression, 9 of which correlated to OS, and a CRC-specific RNA network was constructed	[49]
274 pts and mouse xenografts	WES, WGS	CNA analysis revealed 3 clusters overlapping with consensus molecular subtypes and high chromosomal instability predicted better response to BVZ combination therapy	[50]
276 pts	224 WES, 97 WGS, 215 RNAseq	24 genes were significantly mutated, some novel (*ARID1A, SOX9, FAM123B/WTX*) Potential drug targets: WNT signaling, β-catenin, IGF2, IGFR, ERBB2, ERBB3, MEK, AKT and mTOR Targetable recurrent CNAs: *ERBB2* amplifications and a novel amplification of *IGF2*	[51]
233 (4,742 from 21 cancer types)	WES	4 novel genes with clear connections to cancer were identified	[7]
230 (9423 from 33 cancer types)	WES	Up to 75% of CRCs harbored drug targets, while 59 novel cancer drivers were identified in the total cohort	[52]
213 pts and cell lines	WGS, ChIP-seq	Functional non-coding point mutations at cohesin binding sites (CBSs) were frequent, similarly to other cancers, putatively driving tumorigenesis	[53]
106 pts plus organoids and xenografts	tNGS, WES, WGS, RNAseq	Models retain genetic and transcriptomic tumor characteristics enabling research for improving therapeutic response prediction	[54]
103 pts	tNGS, WES	20 new recurrently mutated genes were identified	[55]

Abbreviations: chromatin immunoprecipitation sequencing (ChIP-seq), colorectal cacner (CRC), copy-number alteration (CNA), gastrointestinal (GI), long non-coding RNA (lncRNA), metastatic tumor (MT), microsatellite instability (MSI), next-generation sequencing (NGS), overall survival (OS), patients (pts), primary tumor (PT), RNA sequencing (RNAseq), targeted NGS (tNGS), whole-exome sequencing (WES), whole-genome sequencing (WGS).

**Table 2:** Multiple biopsy next-generation sequencing dissecting tumor heterogeneity.

Patients (Samples)	Technology	Findings and Potential Clinical Implications	Ref.
88 pts (46 matched PT and MTs and 42 non-metastatic PTs)	WES	Computationally calculated tumor heterogeneity was highly variable, with 70% sub-clone consistency between PT and LM, while high heterogeneity correlated to worse outcomes	[56]
69 pts (Matched PT and MT samples)	tNGS (WGS on 4)	*KRAS*, *NRAS*, and *BRAF* mutations were 100% consistent and recurrent alterations were highly similar, suggesting that NGS of either PT or MT could suffice	[57]
27 pts (97 samples from PT and MTs and 68 samples from a single PT)	tNGS (100 gene panel)	Inter- and intra-tumor variability was due to CNAs, which were highly discordant between PT and MT	[18]
23 (118 MR tissue samples from matched PT and MTs)	WES	Although extensive inter- and intratumor heterogeneity was identified, matched PT and MTs were highly concordant for driver mutations, suggesting the early acquisition of aggressive alterations responsible for metastasis, while the modeof tumor evolution and sub-clonality correlated with disease stage	[58]
18 (Matched PT and LM samples)	tNGS	79.3% of SNVs in the PT were detected in the LM, while 81.7% of LM mutations were found in the PT, suggesting linear progression	[59]
18 (Matched PT and MT samples)	tNGS	While concordance was 93.5%, most tumors showed at least one discordance due to co-evolution, suggesting that sampling over therapy could be useful	[60]
17 (213 matched PT, LN and MT)	Polyguanine-repeat analysis	In 65% and 35% of cases, LN and distant metastases originated from distinct and single PT subclones respectively	[61]
14 pts (70 MR samples from PT and matched liver and/or lung MTs)	tNGS	*RAS* status was preserved in MTs, while emerging mutations in other genes were also identified	[62]
12 (Matched PT and MT)	WGS	15% and 19% of mutations were PT- and MT-specific respectively, while late metastasis is supported Recurrent non-coding mutations: ncRNAs *RP11-594N15.3, AC010091, SNHG14*, 3’ UTRs of *FOXP2, DACH2, TRPM3, XKR4, ANO5, CBL, CBLB* MT-specific oncotargets: *FAT1, FGF1, BRCA2, KDR,* and *AKT2*-, *AKT3*-, and *PDGFRA-3*’ UTRs	[63]
10 early CRC (53 MR samples)	MR-WES	This study supports a shift from Darwinian to neutral evolution during CRC progression	[64]
9 (75 MR PT and 2 LM samples)	MR-WES	All cancers exhibited high ITH due to neutral evolution and drug resistance was attributed to pre-existing minor subclones	[65]
6 (3-5 biopsies per patient)	MR-WES, RNAseq	Although ITH was universal, transcriptomics-guided classification could be independent of ITH	[66]
5 pts (35 MR PT and LM samples)	MR-WES of the PT and MT	Branching evolution was identified, with prevalent CNA-based ITH as a putative source of metastasis	[67]
4 (23 MR PT and MT samples)	MR-WES	Significant inter- but limited intra-patient variability was identified MTs had lower ITH than PTs, while polyclonal seeding was detected	[68]
2 (36 spatiotemporal PT and MT samples)	WES	Different modes of evolution and metastatic progression were identified, depending on the immune microenvironment of the metastatic site Distinct MTs showed different clinical, genomic and immune features An immunoediting score was developed and correlated to immune response and prognosis	[69]

Abbreviations: colorectal cancer (CRC), copy-number alteration (CNA), intra-tumor heterogeneity (ITH), liver metastasis (LM), lymph node (LN), metastatic tumor (MT), multi-regional (MR), next-generation sequencing (NGS), non-coding RNA (ncRNA), patients (pts), primary tumor (PT), RNA sequencing (RNAseq), single-nucleotide variant (SNV), targeted NGS (tNGS), whole-exome sequencing (WES), whole-genome sequencing (WGS).

**Table 3:** Next-generation sequencing of circulating cell-free DNA: clinical utility.

Patients (Samples)	Technology	Findings and Potential Clinical Implications	Ref.
**Static cf/ctDNA next-generation sequencing analysis**			
21,807 (>50 advanced cancer types)	tNGS	Driver gene cfDNA mutation profiles were similar to tumor NGS, while differences were attributed to clonal evolution over therapy leading to resistance	[70]
1422 (sub-study, 21 tumor types)	tNGS, WGS, WGBS	Sensitivity for 12 cancers including CRC was 76% and 74% for stage I-III CRC	NCT02889978 [71]
1397 (advanced CRC)	tNGS	Mutation frequencies in ctDNA were similar to tissue, and multiple distinct resistant mutations were identified in single patients	[19]
1005 (8 cancer types)	CancerSEEK	Sensitivity was 65% and stage-dependent for CRC, suggesting the need for improvement before clinical applicability	[72]
100 (TARGET study, diverse advanced cancers, 23 CRC)	tNGS	Druggable mutations were identified in 41/100 pts, 11/41 received matched therapy and all 11 achieved PR or stable disease	[22]
80 pts	WGS	Recurrent CNVs were identified in multiple chromosomal regions and correlated with stage and prognosis	[73]
**Consecutive liquid biopsies before and after systemic therapy**			
261 (ASPECCT study, plasma samples before and after panitumumab)	tNGS	Baseline high *RAS* mutant allele frequency and EGFR pathway mutations were adverse prognostic factors, while tumor mutational burden increased over time This study suggests potential utility for primary and secondary decision-making	[74]
238 (ASPECCT study, plasma samples before and after panitumumab)	tNGS	79% of baseline samples were WT and 21% mutant *RAS* (associated with worse outcomes), while 32% of baseline-WT tumors had emergent *RAS* mutations	[75]
53 (159 serial samples over chemotherapy)	tNGS	Mutational concordance between tumor and cfDNA was 92.3%, while cfDNA levels were predictive of clinical response	[76]
39 various metastatic cancers, 12 CRC (159 total serial samples over targeted therapy)	tNGS	Monitoring of plasma mutation allele identified potential clonal responses to targeted therapy associated with progression, suggesting potential prognostic and predictive utility	[77]

Abbreviations: cell-free DNA (cfDNA), circukating tumor DNA (ctDNA), colorectal cacner (CRC), next-generation sequencing (NGS), partial response (PR), targeted NGS (tNGS), whole-genome bisulfite sequencing (WGBS), whole-genome sequencing (WGS), wild-type (WT).

**Table 4:** Dynamic emergence of tumor heterogeneity and metastasis: clinical implication of intra-patient heterogeneity.

Patients (Samples)	Technology	Findings and Potential Clinical Implications	Ref.
100 (Matched PT and plasma samples after anti-EGFR)	BEAMing, tNGS	Resistant circulating mutations were detected (*KRAS*, *NRAS*, *MET*, *ERBB2*, *FLT3*, *EGFR*, *MAP2K1*), while treatment cessation led to re-emergence of sensitivity	[78]
83 diverse advanced cancers (14 CRC, Static PT and ctDNA)	tNGS, ctDNA-tNGS	30% of pts achieved disease control and targeting of more drug targets correlated with significantly favorable clinical outcomes, supporting individualized drug combinations	NCT02534675 [20]
47 (archived PT, double MT samples at baseline, PR and progression and serial plasma samples)	tNGS, cfDNA-tNGS	50% of tumor *RAS*-WT patients harbor *RAS* mutations in baseline cfDNA Dynamic tissue and liquid biopsies could predict primary and acquired cetuximab resistance and progression	NCT02994888 [17]
33 (Serial liquid biopsies over HER2 blockade and diverse PT and MT samples)	WES, ctDNA-tNGS	*ERBB2*, *RAS* and *PIK3CA* mutations correlated to HER2-targeted therapy resistance and liquid biopsies identified primary resistance with >85% sensitivity, suggesting utility for decision-making	[79]
22 (archived and post-progression tissue after anti-EGFR and static ctDNA)	tNGS	*RAS* mutations and *HER2/MET* amplification were the most prominent mechanisms of resistance in both tissue and ctDNA, suggesting utility for decision-making	[80]
12 (Matched PT, MT and plasma samples)	tNGS	Limited concordance between ctDNA and PT/MT was identified, suggesting the need for refinement	[81]
7 (diverse tumor samples over anti-EGFR, matched ctDNA, mouse xenografts)	WES, WGS, CNA, BEAMing	*MET* amplifications within rare pre-existing subclones confer resistance in *KRAS*-WT tumors during anti-EGFR therapy	[82]

Abbreviations: beads-emulsion-amplification-magnetics (BEAMing), cell-free DNA (cfDNA), circulating tumor DNA (ctDNA), colorectal cancer (CRC), copy-number alteration (CNA), metastatic tumor (MT), next-generation sequencing (NGS), partial response (PR), patients (pts), primary tumor (PT), targeted NGS (tNGS), whole-exome sequencing (WES), whole-genome sequencing (WGS), wild-type (WT).

**Table 5:** Cell-to-cell heterogeneity and drug response prediction.

Patients/Samples	Technology	Findings and Potential Translational Implications	Ref.
12 pts (1,900 single cells and bulk multi-regional PT and MT)	Multiomics including single-cell Trio-seq and bulk MR-WGS	Several cellular genetic subclones were identified with PTs featuring more extensive subclonality than MTs Single-cell multiomics can track tumor dynamics during progression and metastasis	[84]
11 pts and 7 cell lines (590 patient-derived and 561 cell line-derived single cells)	Single-cell RNAseq and RCA algorithm	Single-cell transcriptomics enabled more detailed sub-classification of CRC subtypes than bulk RNAseq, correlating to prognosis	[85]
3 pts (Single cell-derived clonal organoids)	tNGS, WGS, RNAseq	All three colorectal cancers contained cells resistant to common drugs, while drug sensitivity was variable even among closely related single cell-derived clones, suggesting late emergence of resistance	[8]
2 pts (6 bulk samples and 336 single cells from CRC, normal epithelium and polyps)	WES, single-cell WES	Adenoma and cancer were monoclonal, albeit with distinct mutational landscapes, with cancers further diversifying into distinct subclones 3 new driver mutations were identified (*OR1B1*, *LAMA1*, *ADCY3)*	[86]
2 pts (360 single-cells and bulk PT and LM samples)	Single-cell tNGS, bulk WES	Monoclonal and polyclonal seeding was identified, while rare cell sub-populations were found to correlate with progression and metastasis, although a late-dissemination model was identified	[87]
1 pt (63 single cells)	WES	Two distinct clones were identified, one major with early *APC* and *TP53* mutations and one minor with *CDC27* and *PABPC1* mutations, highlighting the ability of single-cell NGS to identify rare mutations	[88]

Abbreviations: colorectal cancer (CRC), liver metastasis (LM), metastatic tumor (MT), multi-regional (MR), next-generation sequencing (NGS), patient (pt), primary tumor (PT), reference component analysis (RCA), RNA sequencing (RNAseq), targeted NGS (tNGS), whole-exome sequencing (WES), whole-genome sequencing (WGS).
